# Oral Health Training Programs for Community and Professional Health Care Workers in Nairobi East District Increases Identification of HIV-Infected Patients

**DOI:** 10.1371/journal.pone.0090927

**Published:** 2014-03-14

**Authors:** Lucina N. Koyio, Wil J. M. van der Sanden, Elizabeth Dimba, Jan Mulder, Nico H. J. Creugers, Matthias A. W. Merkx, Andre van der Ven, Jo E. Frencken

**Affiliations:** 1 Ministry of Public Health and Sanitation, Department of Primary Health Care Services, Nairobi, Kenya; 2 Radboud University Nijmegen Medical Centre, Department of Global Oral Health, College of Oral Sciences, Nijmegen, The Netherlands; 3 University of Nairobi Dental Teaching Hospital, Department of Oral and Maxillofacial Surgery, Nairobi, Kenya; 4 Radboud University Nijmegen Medical Centre, Department of Oral Rehabilitation, College of Oral Sciences, Nijmegen, The Netherlands; 5 Radboud University Nijmegen Medical Centre, Department of Oral and Maxillofacial Surgery, Nijmegen, The Netherlands; 6 Radboud University Nijmegen Medical Centre, Department of General Internal Medicine, Nijmegen Institute for Infection, Inflammation and Immunity, Nijmegen, The Netherlands; Alberta Provincial Laboratory for Public Health/University of Alberta, Canada

## Abstract

**Background:**

Better knowledge and skills for diagnosis and management of human immunodeficiency virus (HIV) related oral lesions by primary healthcare workers (PHWs) may increase recognition of HIV-related oral lesions (HROLs) and may improve implementation of HIV testing in Kenya. For this purpose training programs at health facility and community level were evaluated.

**Design and Methods:**

A pre-post control-test group design in two administrative divisions of Nairobi East District was used. Clinical competencies of PHWs (n = 32 intervention, and n = 27 control) at health facility level were assessed 9 months after training, and after 6 months for community health workers, (CHWs) (n = 411 intervention and n = 404 control) using written questionnaires, clinical data and patient interviews. Effects on referral for HIV testing and actual HIV testing were assessed by comparing laboratory registries pre- and post training.

**Results:**

PHWs in intervention (n = 27; 84%) and control (n = 15; 60%) divisions, and CHWs in intervention (n = 330; 80%) and control (189; 47%) divisions, completed all questionnaires. Trained PHWs significantly increased their knowledge of HROLs (p<0.02), frequency of oral examinations, diagnosis of HROLs and referral of patients with HROLs for HIV testing. Trained CHWs significantly gained knowledge about HROLs (p<0.02) and referred more patients with HROLs to health facilities. Overall percentage of HIV-positive test results was three-fold for HROLs compared to non-HROLs. Specifically, 70% of patients with oro pharyngeal candidiasis (OPC), the most commonly diagnosed HROL, were confirmed as being HIV-positive. Increase in overall HIV testing rates (1.6% pre-, 1.2% post training) and overall percentage of HIV-positive results (13% pre-, 16% post-intervention) was not significant.

**Conclusion:**

Training programs significantly increased PHW and CHW knowledge, recognition and management of HROLs but increased neither overall HIV testing rates nor overall percentage of positive tests. Speculation is that the health system and patient-related barriers seriously limit HIV testing.

**Trial Registration:**

Netherlands Trial Register NTR2627 (date registered 22nd November 2010), and NTR2697 (date registered 13th January 2011).

## Introduction

Healthcare providers working in a human immunodeficiency virus (HIV) endemic area are often faced with orofacial lesions that are commonly associated with HIV infection [20]. Confirmation of HIV infection has been described in up to 98% of patients with HIV-related oral lesions (HROLs), the most common being oropharyngeal candidiasis (OPC) [1]. OPC is not only an indicator of (early) HIV infection but may also function as a warning sign of immunological and virologic failure in patients on highly active antiretroviral therapy (HAART) [2], [3], [4]. Thus, recognition of HROLs is important for patient care as well as from a public health perspective.

Patients presenting with HROLs at health facilities might not be identified as high-risk patients and consequently not be tested for HIV infection. This may result from inadequate oral health knowledge of professional health workers (PHWs), who are mainly clinical officers (COs), nurses and community health workers (CHWs) [5], [6]. In Kenya, PHWs in health facilities collaboratively work with CHWs to provide primary health care to the community [7]. Different strategies may be implemented in Kenya to improve oral health knowledge and subsequent HIV testing. In order to increase referrals for HIV testing, a training program focused on improving knowledge and skills of PHWs in identification and management of HROLs was implemented in combination with a training program aimed at improving recognition of common HORLs by CHWs at community level.

The primary objective of the present study was to analyze the effects of both training programs [8], [9] in Nairobi East district in Kenya aimed at increasing the knowledge and competence of health care providers in the recognition of HROLs. Secondary objectives were to explore: 1) whether the implementation of these training programs would increase oral examinations by PHWs; 2) whether more patients with suspected HIV infection would be referred from the community to the health facilities; 3) whether overall HIV testing and the capacity to identify high risk patients would increase.

## Materials and Methods

### Study Design

This pre-post control group study design [10] was administered as a: a) retrospective clinical records study and; b) prospective controlled study. The study covered two administrative divisions of Nairobi East District. Randomization Software EASY RA1 Easy Randomizer Version 4.1, State University of Michigan, USA was used and Njiru was randomly designated as the intervention division and Makadara as the control area. According to health records, these two divisions were comparable in terms of population numbers, staff numbers, personal and background characteristics of the staff and workloads at the health facilities. Each division comprised four health facilities. The study included all PHWs (n = 32 in intervention and n = 25 in control division) and all CHWs (n = 411 and 404 in the intervention and control division, respectively). Flow of the participants, as well as activities and types of data that were collected at various stages of the study, are described below and illustrated in Boxes S1 and S2.

### Improvement of Knowledge in Recognition of HROLs by Health Care Providers

Radboud University Nijmegen Medical Center, Department of Global Oral Health and the University of Nairobi Dental School collaboratively developed two one-day training programs, to equip the health workers with basic knowledge and skills for recognition and management of (HIV-related) oral lesions, as well as reminding programs, as described elsewhere [8], [9].

PowerPoint presentations contained simplified text and selected photographs of common (HIV-related) oral lesions (pseudo membranous candidiasis, erythematous candidiasis, angular cheilitis, oral hairy leukoplakia, necrotizing ulcerative gingivitis, Kaposi’s sarcoma, parotid enlargement, herpes zoster, dental caries, periodontitis, dental fluorosis) [1], [3], [11] to enhance healthcare provider ability to recognize these lesions. During the training, PHWs also received practical training, involving HIV-infected patients with HROLs, to improve their clinical skills. In the first three months after the training, PHWs were visited and reminded to review patients with questionable lesions and use PowerPoint presentations containing photographs of HROLs to update their knowledge. Barrier factors affecting their performance were also assessed. All health centers received monthly telephone reminders about the correct use of routine recording tools.

CHWs received an additional module on communication skills and use of educational brochures and posters, to enable them to do community mobilization.

### Pre- and Post-training Assessments

A baseline written assessment was done, to analyse background characteristics and baseline knowledge amongst PHWs and CHWs in the intervention and control divisions, as described elsewhere [5]. On the one hand, oral health care tasks are relatively new among professional primary health care providers as this topic is neither emphasised during pre-service nor in-service training. On the other hand, low literacy levels in this community and lack of formal medical training among CHWs explains the numerous traditional beliefs and practices as well as misconceptions regarding HIV related (oral) diseases. To gather as much information as possible from both health workers and the CHWs, open ended questions were additionally used. The PHW questionnaire covered knowledge of symptoms of HROLs, the clinical appearance of HIV-suspected conditions, general dental knowledge, knowledge of OPC, common appearances of OPC, knowledge of periodontitis, causes of dental caries, past training in oral health topics and clinical experiences (Table 1). The 5 domains in the CHW questionnaire were: general oral health knowledge, knowledge of HROLs, opinions regarding oral health problems, encounter of HROLs in the community and current care at community level (Table 2). Both questionnaires were in English. The CHW questionnaire had an additional translated version, in their local language, to enable them to express their views accurately. General results of this assessment have been presented elsewhere [5], [6]. These questionnaires were again administered; 9 months after the training for PHWs and 6 months after training for CHWs (post-training assessment). Training and evaluation of PHWs was performed between February 2010 and November 2010. Thereafter, CHW training was done and evaluated (February 2011 to September 2011).

**Table 1:** Pre- and post-training single item questions and mean domain scores, standard deviations (sd), p-values and maximum scores in the PHW questionnaire for intervention and control division.

Domain		pre test^1^			post test^2^			
	group	mean	sd	p	mean	sd	p	max
**Knowledge on HIV-related oro facial diseases**								
Symptoms of HROL*	I	4.44	1.93	0.70	5.37	1.18	0.0002	**7**
	C	4.50	1.58		3.93	1.28		
Clinical appearance of HIV-suspected conditions*	I	1.89	0.89	0.18	2.15	0.53	0.0001	**3**
	C	1.47	1.06		1.4	1.06		
Knowledge of OPC*	I	1.63	1.22	0.81	2.41	0.64	0.0096	**3**
	C	2.13	0.92		1	0.93		
Common appearances of OPC	I	1.04	0.44	0.61	2	0.48	<.0001	**2**
	C	0.93	0.7		1	0.85		
**Knowledge on general oral diseases and conditions**								
General dental knowledge*	I	2.91	1.53	0.12	4.72	1.24	0.0019	**6**
	C	2.13	1.47		2.93	1.79		
Knowledge of periodontitis	I	1.56	1.15	0.26	2.78	0.51	0.0003	**3**
	C	1.13	1.13		1.8	1.15		
Causes of dental caries	I	0.81	0.56	0.05	2.67	1.78	0.0128	**3**
	C	1.4	0.99		1.27	0.88		

1 Intervention group (I): n = 32; Control group(C): n = 25.

2 Intervention group (I): n = 25; Control group(C): n = 15.

HROLs – HIV related oral lesions.

*domains.

**Table 2:** Pre- and post-training mean domain scores, standard deviations (sd), maximum scores and p-values in the CHW questionnaire for intervention and control divisions.

Domain		pre test^1^			post test^2^			
	group	mean	sd	p	mean	sd	p	max
General oral health knowledge	I	0.49	0.16	0.11	0.76	0.13	<.0001	1
	C	0.47	0.12		0.48	0.13		
Knowledge of HROLs	I	0.43	0.17	0.81	0.65	0.15	<.0001	1
	C	0.44	0.16		0.47	0.17		
Opinions in oral health problems	I	0.82	0.22	0.56	0.94	0.15	0.015	1
	C	0.79	0.23		0.9	0.18		
Encounter of HROLs in the community	I	1.97	0.59	0.84	1.85	0.52	0.0001	4
	C	1.98	0.59		2.07	0.56		
Current care at community level	I	0.63	0.21	0.11	0.72	0.18	<.0001	1
	C	0.6	0.2		0.6	0.19		

1 intervention group (I): n = 32; control group(C): n = 27.

2 intervention group (I): n = 27; control group(C): n = 15.

HROL – HIV related orofacial lesions.

### Performance of oral Examinations and Identification of HROLs by PHWs

PHWs routinely record outpatient consultation details, including oral pathologies (dental decay, periodontal diseases, malocclusion and (HIV-related) oral mucosal lesions) in daily tally sheets and outpatient registers. These indicators were retrospectively and manually extracted from the clinical records from the year prior to the training as well as after training, in the intervention and control divisions. Clinical records were studied in assessing whether or not an oral examination had been performed. This was subject to limitations such as: failure of PHWs to record oral examinations if they did not find relevant oral pathology; some orofacial lesions such herpes zoster had been diagnosed without oral examination. Therefore, additional assessments were done by structured exit interviews (n = 924) in the intervention area and (n = 666) control division, a month before the training and thereafter on patients leaving the health facilities, to assess whether an oral examination had been performed by PHWs (Box S1).

### Referral by CHWs of Patients from the Community with HIV-related Oral Lesions

At recruitment, all CHW and PHWs had been introduced to their roles: educating the community, referral of patients to PHWs for further care and use of the government- owned CHW service delivery log book (CHWSDLB) for recording all referrals that they made from the community. The CHWSDLB was modified, through addition of two columns for the capture of data on the number of referred HROLs. CHWs received short messages on their mobile phones, known to be effective reminders in this setting [12], and supervisory visits to remind them to record and submit monthly data from the community through community health extension workers. Data were collected two months before the aforementioned CHW training program and throughout the succeeding 6 months after the training.

### HIV Testing Rates and Capacity to Identify High Risk Patients

The expectation was that: a) trained PHWs would refer more patients with HROLs from outpatient consultation rooms to the laboratory for HIV testing and; b) trained CHWs would refer more patients with HROLs from the community to the PHWs and consequently further increase the number of patients with HROLs referred to the laboratory for HIV testing. This would increase overall HIV testing rates. PHWs refer patients to the laboratory within the same health facility for HIV testing. There a rapid HIV diagnostic test is done. All laboratories at each health facility keep records including the number and results of the tests. However, reasons for referral are routinely not included. The latter, including specific types of HROLs referred, were included after the training. Routine HIV laboratory data from voluntary counseling and testing (VCT) clinics, from prevention of mother to child transmission of HIV infection (PMTCT) and from diagnostic counseling and testing (DTC) in tuberculosis clinics were not part of the present analysis. In cases where patients with HROLs were referred by PHWs from these clinics to the laboratory for testing, the data was captured in the laboratory register.

### Data Analysis

Data were checked for completeness and consistency before entry into Excel files. Data analyses were conducted, using SAS software (version 9.2, SAS Institute, Cary, NC, USA) by JM (biostatistician at Radboud University Nijmegen Medical Centre). Background characteristics were described, using frequency and descriptive statistics. Chi-square tests were used in comparing differences in percentages. Pre-test means were analyzed, using Student-T-tests, whereas a multiple regression model with correction for pre-test values was used for analyzing post-test means.

For all tests, a significance level of 0.05 was used. For effect assessment the data listed below were compared in both divisions, before and after the PHW and CHW trainings.

### Study Participants

In both divisions, and for both test and control groups, student-T-Tests were used to compare baseline knowledge of lost-to-follow-up PHWs and CHWs with that of PHWs and CHWs who completed the post-training questionnaire.

### Assesment of Knowledge in Recognition of HROLs by Health Care Providers

A panel of two experienced dentists and native speakers of both languages independently coded and iteratively harmonized open-ended data in the PHW and CHW post-study written assessments, as no prototype questionnaire was available and as much information was required to make a tailor-made responsive training programme. To generate an initial codebook for open-ended questions, the panel discussed to reach consensus on correct and wrong responses for open ended questionnaires with multiple responses. To improve the code book the scope of right and wrong answers was further defined using the questionnaire responses. The coders identified and discussed unsatisfactory responses, partially answered questionnaires, ambiguous statements as well as wordings of correct responses. Codes were assigned to agreed correct responses, wrong responses, unanswered questions and ‘I do not know’ responses. The number of unanswered questions was negligible since the respondents had the ‘I do not know’ option. In addition research assistants checked for completeness of the scripts during questionnaire administration. For analysis, unanswered questions and ‘I do not know’ questions were regarded as lack of knowledge and were therefore considered as wrong responses. The two open ended questions that had no right or wrong answer, as that they needed ‘further explanation’ were analysed separately. After coding the first seven scripts, results were checked for agreements and differences. Disagreements were discussed until consensus was reached; 90% agreement between the two coders permitted reliable coding of the remaining transcripts.

Data were analysed in line with the aforementioned domains. After correction for baseline differences, pre- and post-training study mean scores for PHWs and CHWs were compared at (p = 0.05). Pre-test means were analyzed through use of Student-T-tests, whereas a multiple regression model with correction for pre-test values was used for analysis of post-test means.

### Assessment of Performance of Oral Examination and Identification of HROLs by PHWs

Percentages were used to compare the difference in proportions of the number of outpatient consultations that included an oral examination, the number of diagnoses of HROLs and the number of outpatient consultations.

### Assessment of CHW Referrals of Patients with HIV-related Oral Lesions from the Community

Numbers of HROLs referred from the community to the health facilities were compared, as well as the numbers of HROLs diagnosed by PHWs in the outpatient clinics.

### Assessment of HIV Testing Rates and Capacity to Identify High Risk Patients

Proportions of a) outpatient consultations that included an HIV test b) HROLs diagnosed in the outpatient clinics that received an HIV test and c) HIV-positive test results in oral and non-oral lesions were expressed as percentages. Chi square tests were used in comparing differences in percentages at p<0.05.

Owing to an HIV test kits supply shortage, this analysis excluded June and July 2010 data.

### Ethical Approval

Kenyatta National Hospital/University of Nairobi Ethics and Research Committee gave ethical clearance (number KNH-ERC/A/474). Ministry of Public Health and Sanitation also gave written approval (Ref. No. MPHS/IB/1/14 Vol. III) to do the study and collaborate with Kenya National AIDS Control Program (NASCOP) and Department of Health Promotion. Nairobi Provincial Director of Public Health and Sanitation and the district head also gave written approval for the study to be carried out in Nairobi East district.

## Results

### Study Participants

Twenty-seven PHWs completed the post-training questionnaire (n = 21 nurses, 6 clinical officers; response rate 84%) in the intervention group, and 15 PHWs (n = 12 nurses, 3 clinical officers; response rate 60%) in control group. Baseline knowledge of PHWs who were lost to follow up in both divisions was similar to that of the PHWs who completed the post-training questionnaire (p>0.1), except that baseline knowledge of symptoms of HROLs of PHWs who were lost in the intervention division was better (p = 0.003).

The CHW post-training questionnaire was completed by 330 CHWs (response rate 80%) in the intervention divisions and 189 CHWs (response rate 47%) in the control area. CHWs who were lost to follow-up in the test group were less knowledgeable about HROLs than those who completed the post-training questionnaire(p<0.025). No differences in knowledge were found between CHWs who were lost to follow-up in the control group and those who completed the post-training questionnaire (p>0.08).

### Effects of Knowledge in Recognition of HROLs by Healthcare Providers

PHWs had low to moderate baseline knowledge regarding (HIV-related) oral health topics. Details of the pre-training results are described elsewhere [5].

The intervention group had significantly higher mean post-training scores than the control group had (p<0.02) in all assessed areas, except for clinical appearance of HIV-suspected conditions, where the difference was marginal (p = 0.17) (Table 1).

The scores for all domains at baseline showed no differences in both CHW groups (p>0.11) and were moderate, except for opinions about performing oral health tasks, where they both scored high. In the post-training assessment, the intervention group had statistically significantly higher mean scores in all domains (p<0.02) (Table 2).

### Effects on Performance of Oral Examinations and Identification of HROLs by PHWs

As shown in Table 3, at baseline, PHWs in the intervention division performed an oral examination in 2.2% (1,349 out of 60,216) of patients who consulted them in outpatients clinics, and diagnosed 0.3% (n = 178) HROLs. PHWs in the control division performed oral examinations in 2.9% (1,643 out of 56,522) of patients who consulted them in outpatient clinics and diagnosed 0.4% (n = 211) HROLs. Post-training data showed a remarkable increase in frequency of oral examinations by the PHWs (n = 2,301 out of 66,072; 3.5%) and a two-fold increase in the percentage of HROLs diagnosed in the intervention group (n = 391 HROLs; 0.6%), frequency of oral examinations (1,303 out of 50,242; 2.6%) and the percentage of HROLs diagnosed by the PHWs in the control group (n = 120 HROLs; 0.2%) remained stable.

**Table 3:** Clinical data from outpatient registers related to: outpatient records examined, consultations which included oral examination, diagnosis made on dental decay, diagnosis made on gum diseases and diagnosis made on suspected oral HIV lesions by PHWs in the intervention and control divisions, expressed in percentages of outpatient consultations.

Month/year		No. of outpatientrecords examined	No. of OPD consultations whichincluded an oral examination	%	No. of diagnosis made onsuspected oral HIV lesions	%	No. of all patients who were testedfor HIV from outpatient clinics	%	
March-Dec 2009^1^	*I*	60,216	1,349	2.2%	171	0.3%	946	1.6%	
	*C*	56,522	1,643	2.9%	201	0.4%	1,842	3.3%	
Training of PHWs									
March - Dec 2010^2^	*I*	66,072	2,301	3.5%	391	0.6%	782	1.2%	
	*C*	50,242	1,303	2.6%	120	0.2%	916	1.8%	
Jan - Feb 2011^3^	*I*	15,026	582	3.9%	121	0.8%	–		
	*C*	10,006	274	2.7%	15	0.1%	–		
Training of CHWs									
March - July 2011^4^	*I*	38,656	1,219	3.2%	188	0.5%	–		
	*C*	31,952	815	2.6%	91	0.3%	–		

*I* = intervention division.

*C* = Control division.

1 pre-training data for PHWs.

2 post-training data for PHWs.

3 pre-trainig data for CHWs.

4 post- training data for CHWs.

OPD = outpatient department.

Interviews of patients leaving consultation rooms before the training program, showed that PHWs performed oral examinations during outpatient consultations in 10% of cases in both divisions. After the training program, frequencies increased two-fold in the intervention division and remained higher than in the control division throughout the observation period. The peak in frequency of oral examinations, of 45% in intervention and 15% in control divisions, was observed between July and August 2010, during the ‘FIFA 2010 World Cup’ national HIV campaign (Box S3).

Diagnosed HROLs consisted of OPC, herpes zoster, bilateral parotidomegaly, mouth ulcers, Kaposi’s sarcoma, periodontal infections and unspecified mouth problems. Oral hairy leukoplakia was not diagnosed (Table 4).

**Table 4:** Clinical data drawn from laboratory registers to compare pre-training (March to December 2009) and post-training (March to December in 2010) performance of professional health workers in the intervention and control divisions, regarding a)number of patients with HIV-related oral lesions(including types of lesions) that were diagnosed b) number of patients with HIV-related oral lesions who were given an HIV test c) percentages of HIV-positive test results for patients with HIV-related (non) oral lesions.

Observation period	Type of lesiontested in thelaboratory	No. of patient s withHROLs diagnosedin OPD	No. of patient Tested for HIV	HIV positive result	% of HIV positive result	No. of patients with HROLs diagnosed in OPD	No. of patient Tested for HIV	HIV positive result	% of HIV positive result
		Intervention division				Control division			
**March to December** **2009**	Total overall referrals	n/a	946	127	13%		1842	268	15%
March to May 2010	HROLs	144	61	21	34%	47	0	0	0%
	Non-oral lesions	n/a	251	22	9%		317	47	15%
August to December 2010	HROLs	171	49	21	43%	48	26	8	31%
	Bilateral parotidomegaly		2	1	50%		2	0	0%
	Herpes zoster^1^		3	2	67%		2	1	50%
	Kaposi’s sarcoma^1^		1	1	100%		5	1	20%
	OPC Adults^1^		23	16	70%		11	2	18%
	OPC children <5 yrs		3	0	0%		0	0	0%
	Mouth ulcers		2	0	0%		2	1	50%
	Unspecified lesions		15	1	7%		4	3	75%
	Oral Hairy Leukoplakia^1^		0	0	0		0	0	0
	Periodontal infections^1^		0	0	0		0	0	0
**Total for March to December 2010**	Total HROLs	315	110	42	38%	95	26	8	31%
	Total non oral lesions		590	62	11%		579	86	15%
	Total overall referrals		700	104	15%		605	94	16%

1 group one HIV-related oral lesions according European Economic Community (EEC) Clearinghouse on oral problems related to HIV infection (1993) criteria.

HROLs = HIV-related oral lesions.

OPC = oropharyngeal candidiasis.

OPD = Outpatients department.

### Effects on Referral by CHWs of Patients from the Community with HIV-related Oral Lesions

In the two months preceding the training program, CHWs in the intervention division referred 26 (1%) HROLs from the community, out of a total of 2,492 referrals that they made to the linking health facilities, while those in the control division referred 44 (0.4%) out of a total of n = 7,913 overall referrals. Over the succeeding 6-month observation period, CHWs in the intervention division increased referrals of HROLs to 646 (2%) out of 30,466 total referrals, while the control division referred 38 (0.3%) out of 14,672. Although the total percentage of referrals could not be reliably calculated because of overlapping general indicators in the CHWSDLB, our results indicate that CHWs in the intervention division referred much more patients with HROLs to the health facilities after having been trained.

### Effects on HIV Testing Rates and Capacity to Identify High-risk Patients

At baseline, the HIV testing rate was 1.6% (n = 946 out of 60,216) in the intervention division and 3.3% (1,842 out of 56,222) in the control division. The difference between the intervention and control divisions was attributed to the presence of a large educational institution in the control area, which was found to have referred many students for medical check-ups that often included an HIV test. HIV testing rates were slightly lower after the PHW training program, both in the intervention division 1.2% (782 out of 66,072) and in the control division 1.8% (916 out of 50,242) (Table 3). Overall, the percentage of HIV-positive test results was 13% in the intervention, and 15% in the control division at baseline (Table 4).

Not all patients with HROLs that were diagnosed at the outpatient clinics presented at the laboratory for HIV testing. In 2010, after the PHW training, 35% (110 out of 315) of patients diagnosed with HROLs were HIV-tested in the intervention division and 27% (26 out of 95) in the control division (Table 4). In both groups patients with periodontal disease were not referred for HIV testing.

The overall percentage of patients with oral lesions who had an HIV-positive test was 38% (42 out of 110) in the intervention division, in comparison to 31% (8 out of 26) in the control division (p = 0.65). When individual HROLs were considered, 43% of patients in the intervention, compared to 31% in the control division, were found to be HIV-sero-positive, (p = 0.33). More specifically, HIV infection was confirmed in 70% of the patients with oropharyngeal candidiasis in the intervention division, but in only 18% in the control division (p = 0.01). Only 20% of patients diagnosed with Kaposi’s sarcoma in the control division showed an HIV-positive test result, indicating that the clinical diagnosis was not correct.

The percentage of HIV-positive test results was three times higher for patients with HROLs than for patients with non-oral lesions (38% for HROLs compared to 11% for non-oral lesions) in the intervention division and two times higher (31%) for HROLs than 15% for non-oral lesions in the control division.

The overall percentage of all HIV-positive test results was 15% in the intervention division and 16% in the control one after the 9-month PHW evaluation period.

## Discussion

This study was performed in communities of high HIV prevalence where the burden of detecting/identifying HROLs is complicated by that fact that the majority of HIV- infected patients are unaware of their HIV status and are therefore not receiving any HIV treatment [13], [14]. In addition, nearly 12,000 HAART patients [9] were under the care of PHWs as well as CHWs who lacked sufficient competences in identifying HROLs. Earlier studies showed that these PHWs and CHWs were willing to include oral healthcare tasks related to HIV patients into their routine practice [6], [15].

### Effects on Knowledge Related to Health Care Provider Recognition of HIV-related Oral Lesions

The training program significantly improved PHWs and CHWs knowledge about (HIV related) oral health topics. This study showed that CHWs could play an important role in educating the community in the clinical importance HROLs and the need to seek early medical care. The marginal gain in knowledge of PHWS about the clinical appearance of suspected HIV oral lesions suggests a need for more emphasis on teaching these healthcare workers about various lesions, in future training programmes. Periodontal diseases need to be particularly emphasised, as none of the diagnosed lesions were referred for HIV testing. Emphasis may also need to be placed on identification of oral hairy leukoplakia as this could be mistaken for OPC.

### Effects on Performance of Oral Examinations and Identification of HIV-related Oral Lesions

An increase in the frequency of examinations as well as in the proportion of HIV lesions diagnosed in outpatient consultations was seen in post-training data, in the intervention division. This was attributed to the increased knowledge. This finding is supported by data derived from patient interviews; from self-reporting in written assessments and from laboratory registers. However, although there was a two-fold increase in the frequency of oral examinations and in diagnosis of HROLs in the intervention division, the frequency was very low for this population with high HIV prevalence.

### Effects on PHW Capacity to Identify High-risk Groups

The overall HIV-positive result for patients with HROLs was up to three times higher than for referrals of patients with non-oral lesions, even when HROLS included unspecified mouth lesions not strongly associated with HIV infection as defined in the 1993 European Economic Community Clearinghouse on oral problems related to HIV infection and WHO Collaborating Centre on Oral manifestations of the immunodeficiency virus [16] criteria. However, the increase in overall HIV-positive results for patients with HROLs did not increase the overall percentage of all HIV-positive tests in either division. OPC, the most common HROL, was highly predictive of HIV infection in the intervention division. The performance of trained PHWs was comparable to that of experienced examiners [1], [17], thereby indicating improvement of practical skills among trained PHWs. The importance of training in practical skills was particularly underlined by the low percentage of positive HIV test results in patients with diagnosed OPC and Kaposi’s sarcoma in the control group. Overall, test results could also be further improved if augmented with good medical history [18]. Although identification of HROLs cannot be considered an HIV screening test oral examination, as a simple and non-invasive procedure it may increase the chance of detecting HIV infection in a person. PHWs also identified other oral conditions such as dental caries and counselled patients on preventive care. Most importantly, PHWs also identified HAART patients with virological failure [19].

### Effects on HIV Testing Rates, Patient Referrals and Perceived Barriers

Overall, considering the high HIV prevalence in the country, HIV testing rates from outpatient clinics remained very low in both divisions. Although trained PHWs increased the frequency of oral examinations and the number of HROLs diagnosed, their performance did not increase HIV testing rates. During reminder visits, their high workload was mentioned by PHWs as a barrier to HIV testing. High workloads averaging to 60–100 consultations per PHW per day, as presented in Table 3, may prevent PHWs from having sufficient consultation time for each patient [20]. PHWs also reported reluctance of patients with HROLs to have an HIV test done. Over 65% of patients diagnosed with HROLs did not get tested for HIV. Refusal of 28% of routine dental patients with HROLs to undergo an HIV test was also reported [1]. PHWs further attributed refusal to undergo HIV testing to the HIV testing point. Because outpatient clinics are busy, patients are routinely referred to the laboratory for HIV testing. Opting out of some patients was due to the long waiting time and to stigma from patients in the waiting bays. It was noted that PHWs failed to refer patients with periodontal diseases, although diagnoses were made. PHWs may also fail to refer patients with HROLs if they do not have the skills for diagnosing the lesions, as observed in the relatively lower number of referrals of HROLs in the control division.

Contrary to expectation, increased referral patients with HROLs by CHWs to the health facilities did not translate to increased PHW identification of oral HIV lesions (Table 3), suggesting that patients did not seek consultations at the health facilities. It was unlikely that they went directly to the VCT clinics, although this was not checked. Resistance to HIV testing is a concern in Kenya, often related to low risk perception and fear of social stigma [13]. In this community additional barriers included misconceptions about HROLs, particularly OPC. Baseline assessment showed that nearly 30% of CHWs related OPC to witchcraft, gastrointestinal disorders, sugary or hot foods and consumption of milk. This often resulted in patients’ seeking traditional remedies.

### Study Limitations

Retrospective clinical data were sometimes incomplete, owing to countrywide shortages and unskilled record clerks. Although all PHWs were included in the two divisions, the sample size for PHWs in the control group was smaller than had been planned for. This was due to nationwide staff shortages. Three months after the PHW training, the government, through the Economic Stimulus Program [21] employed additional (n = 17) PHWs in the intervention and (n = 10) PHWs in the control division, who may have diluted the effect of the intervention division as seen after July 2010 (Box S3). In the control division, there was a big fire accident during the CHW evaluation [22], leading to massive displacements of CHWs and the low post-training response rate. The observed peak in oral examination frequency in both divisions (Box S3) in June 2012 coincided with a nationwide HIV campaign termed ‘The FIFA World Cup HIV campaign’, which led to a massive increase in community mobilization during the observation period. The other limitation was caused by nationwide shortages in supply of HIV test kits in June and early July 2010, which lowered the total number of HIV tests for 2010. Towards the end of 2010, the government introduced new laboratory regulations (for HIV testing and counseling (HTC)) that made it difficult to identify HIV tests that were performed in outpatient clinics in 2011 (Table 4). This being an early evaluation of the implementation project, the results presented may only in part reflect the actual effect of the project.

## Conclusion

The competence of PHWs and CHWs in Nairobi East district in diagnosing and managing HROLs is insufficient. Training programs significantly improved knowledge of PHWS and competences in their clinical performance regarding routine oral examination and identification of HIV-high-risk patients but did not significantly increase overall HIV testing rates. Training of CHWs significantly increased their knowledge of HROLs and their referrals of community members with HROLs to a health facility but referrals from community did not significantly increase the number of HROLs diagnosed in the health facilities. The speculation is that health system barriers seriously limit HIV testing. Health system related barriers particularly high workloads among health providers seriously hindered HIV testing in outpatient clinics. The alternative of referring of patients to the laboratory for HIV tests was both stigmatizing and time consuming to the patients leading to ‘loss’ of patients. Patient-related barriers such as low HIV risk perception, fear of discrimination and misconceptions about (HIV related) oral diseases further contributed to patients opting out from HIV testing. Health workers related barriers which included insufficient knowledge and low risk perception of HIV related oral diseases could be addressed through emphasis on oral health topics during the pre-service and in-service training as well as support supervision of the health care workers. Interventions to integrate oral health care of HIV patients into the primary healthcare system should address these barriers.

## Supporting Information

Box S1 **Flow diagram of study design and of participants (PHW).** Flow diagram of study design and flow of participants (professional health workers in health facilities) through all stages of implementation and evaluation of the training program (according to CONSORT 2010 Flow Diagram).
(TIF)

Box S2 **Flow diagram of study design and of participants (CHW).** Flow diagram of study design and flow of participants (community health workers) through all stages of implementation and evaluation of the training program (according to CONSORT 2010 Flow Diagram).
(TIF)

Box S3 **Frequency of oral examinations.** Frequency of oral examinations expressed as a percentage of outpatient consultations by professional health workers.
(TIF)

## References

[1] Agbelusi GA, Wright AA (2005) Oral lesions as indicators of HIV infection among routine dental patients in Lagos, Nigeria. Oral Diseases 11: 370–373 doi:10.1111/j.1601-0825.2005.01132 16269028 10.1111/j.1601-0825.2005.01132.x

[2] Anver M, Opinya G, Hussein A (2010) HIV related oral manifestations of children and adolescents aged 2–15 years living in Nairobi and Mombasa. J Kenya Dent Ass 1: 93–97.

[3] Hamza OJM, Matee Mecky IN, Elison NM S, Kikwilu E, Mainen J M, et al (2006) Oral manifestations of HIV infection in children and adults receiving highly active anti-retroviral therapy (HAART) in Dar es Salaam. BMC Oral Health 6: 12. 16916469 10.1186/1472-6831-6-12 PMC1559688

[4] Miziara ID, Weber R (2008) Oral lesions as predictors of highly active antiretroviral therapy failure in Brazilian HIV-infected children. J Oral Pathol Med 2008 37: 99–106. 10.1111/j.1600-0714.2007.00598.x 18197855

[5] Koyio LN (2014) Knowledge of primary health care providers regarding HIV-related oral facial, and other, common oral diseases and conditions. In: Koyio LN, thesis. Oropharyngeal candidiasis as a marker for HIV infection: impact of community and professional health care workers in Kenya. Nijmegen: Radboud University Nijmegen. 59–72.

[6] Koyio LN (2014) Knowledge of Nairobi East District Community Health Workers on HIV related oral facial lesions and other common oral lesions. In: Koyio LN, thesis. Oropharyngeal candidiasis as a marker for HIV infection: impact of community and professional health care workers in Kenya. Nijmegen: Radboud University Nijmegen. 93–108.

[7] MoH (2006) Taking the Kenya Essential Package for Health to the community. A Strategy for the delivery of level one services. Ministry of Health, 2006. Available: http://marsgroupkenya.org/pdfs/2011/01/Ministry_PDFS/Ministry_of_Public_Health_and_Sanitation/Documents/Taking_the_Kenya_Essential_Package_for_Health_to_the_Community.pdf.

[8] Koyio LN, van der Sanden WJM, van der Ven A, Creugers NHJ, Merkx MAW, et al (2013) A community-based oral health promotion model for HIV patients in Nairobi East District in Kenya: a study protocol. Journal of Public Health Research, 2013 2: e5. 10.4081/jphr.2013.e5 PMC4140327 25170476

[9] Koyio LN, van der Sanden WJM, van der Ven A, Nico Creugers N, Merkx MAW, Frencken JE (2012) Study protocol: Effect of education of primary health care workers on HIV-related oral lesions in Nairobi East district. Journal of Public Health Research 1: 137–140. 25181248 10.4081/jphr.2012.e20 PMC4140363

[10] Zwarenstein M, Treweek S, Gagnier JJ, Altman DG, Tunis S, et al (2008) Improving the reporting of pragmatic trials: an extension of the CONSORT statement. BMJ 337: a2390. 19001484 10.1136/bmj.a2390 PMC3266844

[11] Ranganathan K, Hemalatha R (2006) Oral Lesions in HIV Infection in Developing Countries: an Overview. Adv. Dent. Res. 19: 63. 10.1177/154407370601900113 16672552

[12] Zurovac D, Sudoi RK, Akhwale WS, Ndiritu M, Hamer DH, at al (2011) The effect of mobile phone text-message reminders on Kenyan health workers’ adherence to malaria treatment guidelines: a cluster randomised trial. Lancet 2011 378: 795–803 doi:10.1016/S0140-6736(11)60783-6 PMCID: PMC3163847 Available: http://www.ncbi.nlm.nih.gov/pmc/articles/PMC3163847/. Accessed 3 August 2013. 10.1016/S0140-6736(11)60783-6 PMC3163847 21820166

[13] KDHS (2008–09). Kenya National Bureau of Statistics (KNBS) and ICF Macro. 2010. Kenya Demographic and Health Survey 2008–09. Calverton, Maryland: KNBS and ICF Macro. Kenya demographic and health survey 2008–09. Available: http://www.unfpa.org/sowmy/resources/docs/library/R313_KNBS_2010_Kenya_DHS_2009_final_report.pdfhttp://nascop.or.ke/aboutus.php. Accessed 20 July 2013.

[14] MoH (2007) Ministry of Health, Kenya. National AIDS and STI Control Programme. Kenya AIDS Indicator Survey 2007: Preliminary Report. Nairobi, Kenya. July 2008. Available: http://www.prb.org/pdf09/kaiskenyadatasheet.pdf. Accessed 20 August 2013.

[15] Koyio LN, Kikwilu E, Mulder J, Frencken JE (2012) Attitude, subjective norms and intention to do routine oral examination for oro-pharyngeal candidiasis as perceived by primary health care providers in Nairobi Province. Journal of Public Health Dentistry 73: 127–134. 22762414 10.1111/j.1752-7325.2012.00353.x

[16] EC-Clearinghouse (1993) European Economic Community (EEC) Clearinghouse on Oral Problems Related to HIV infection and WHO Collaborating Centre on Oral Manifestation of Human Immunodeficiency Virus. Classification and diagnostic criteria of oral lesions in HIV infection. J. Oral Pathol. Med 22: 289–291. 8229864

[17] Bhayat A, Yengopal V, Rudolph M (2010) Predictive value of group I oral lesions for HIV infection. Oral Surg Oral Med Oral Pathol Oral Radiol Endod 109: 720–723. 20303299 10.1016/j.tripleo.2009.11.019

[18] Robinson PG, Challacombe SJ. Sheiham A (1998) Oral examination: a screening tool for HIVinfection? Sex Transm Inf 74: 345–348. 10.1136/sti.74.5.345 PMC1758150 10195030

[19] Koyio LN (2014) High level drug resistance in patients on chronic antiretroviral treatment presenting with oropharyngeal candidiasis in Kenya. In: Koyio LN, thesis. Oropharyngeal candidiasis as a marker for HIV infection: impact of community and professional health care workers in Kenya. Nijmegen: Radboud University Nijmegen. 135–146.

[20] Ferrinho P, Sidat M, Goma Fand Dussault G (2012) Task-shifting: experiences and opinions of health workers in Mozambique and Zambia. Human Resources for Health 10: 34–41. 22985229 10.1186/1478-4491-10-34 PMC3515799

[21] KESP (2010) Kenya Economic Stimulus Program: Available: http://en.wikipedia.org/wiki/Kenya_Economic_Stimulus_Program.Accessed 13 August 2013.

[22] Kenya (2011) Kenya: The Sinai Slum Fire Tragedy – Kenya Pipeline Issues Warning.: Available: http://allafrica.com/stories/201109140129.html. Accessed 25 April 2013.

