# Supplementary figures and images for: Oral Health Training Programs for Community and Professional Health Care Workers in Nairobi East District Increases Identification of HIV-Infected Patients
Source: PLoS One. 2014 Mar 14;9(3):e90927. doi: 10.1371/journal.pone.0090927 (PMC3954587; doi:10.1371/journal.pone.0090927)

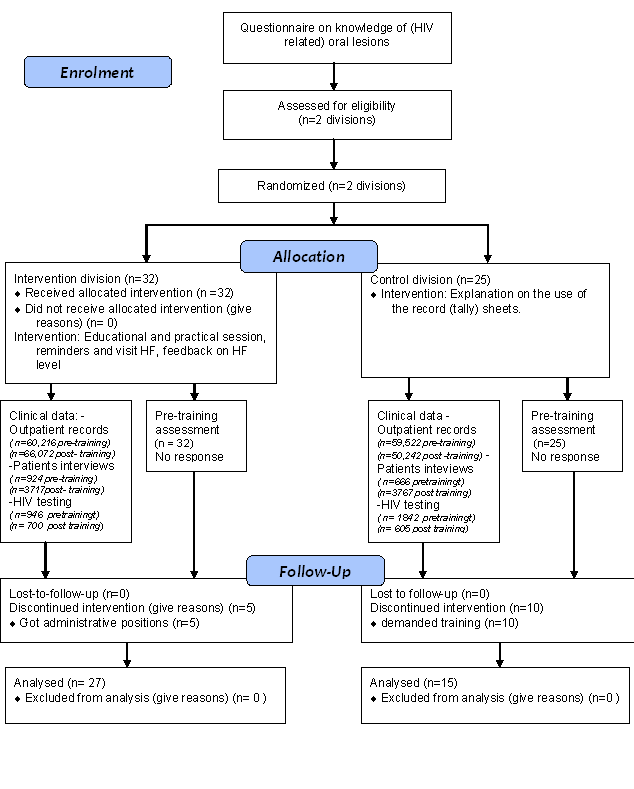

Supplement: Box S1 — Flow diagram of study design and of participants (PHW). Flow diagram of study design and flow of participants (professional health workers in health facilities) through all stages of implementation and evaluation of the training program (according to CONSORT 2010 Flow Diagram). (TIF) [file pone.0090927.s001.tif]

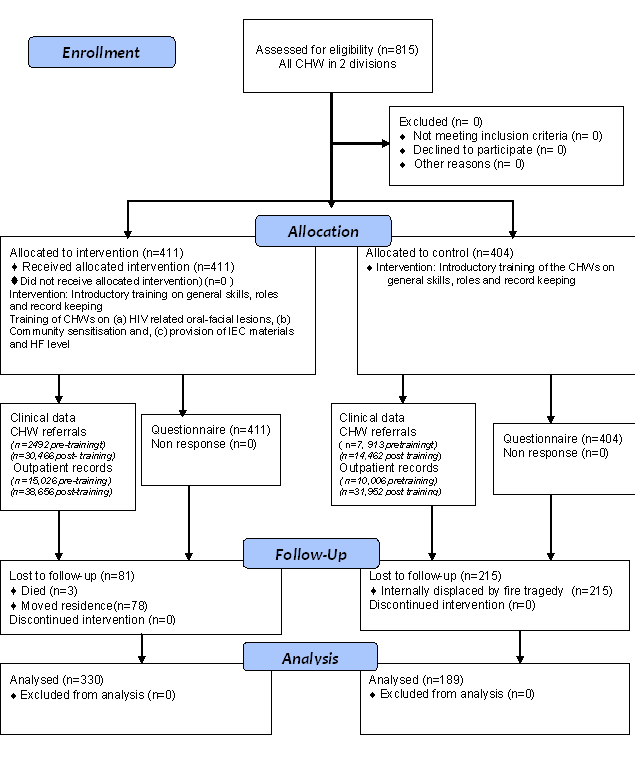

Supplement: Box S2 — Flow diagram of study design and of participants (CHW). Flow diagram of study design and flow of participants (community health workers) through all stages of implementation and evaluation of the training program (according to CONSORT 2010 Flow Diagram). (TIF) [file pone.0090927.s002.tif]

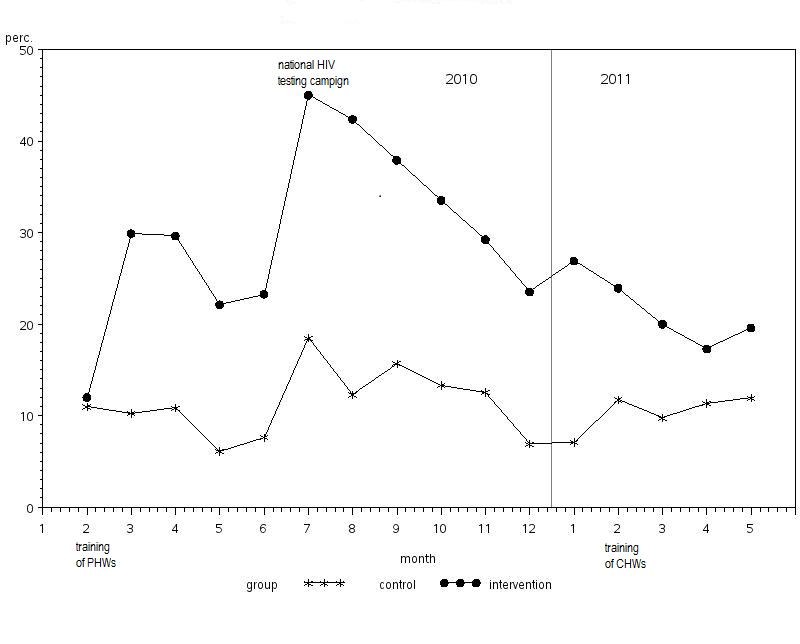

Supplement: Box S3 — Frequency of oral examinations. Frequency of oral examinations expressed as a percentage of outpatient consultations by professional health workers. (TIF) [file pone.0090927.s003.tif]
